# A Warning to Non-Panel Men

**Published:** 1914-02-07

**Authors:** W. E. Barton

**Affiliations:** Member W. and D. Non-Panel A. and N.M.U.


### A Warning to Non-Panel Men.

*To the Editor of* The Hospital.

Sir,—In case some of the non-panel men have not read the enclosed report, stating that a circular is going to be sent to them to try and persuade them to join the panel, I think you would be doing us a great service if you printed it in your next issue. It seems to me that it is only a dodge of the London Insurance Committee to get a few more men to join the panel, notwithstanding that at the same meeting satisfaction was expressed at the number of men on the panel, and that Mr. Dawes, the chairman, made use of his well-known "parrot cry" that the doctors were still coming on.

Although I do not agree with Dr. H. H. Mills that many doctors had not come on the panel because they were afraid of having a number of people assigned to them, and do not think he will be so gratified as he expects to be with the result of this circular, it is perhaps as well to warn the non-panel men to be careful not to be caught in this new trap which has been laid for them.—Yours faithfully, W. E. Barton, Member W. and D. Non-Panel A. and N.M.U.

*Enclosure.*

At a meeting of the London Insurance Committee on January 22 the Medical Benefit Sub-Committee reported that in the case of practitioners who undertook the treatment of a limited number of persons new agreements had been modified by the omission in those cases of the clause requiring practitioners to treat persons who might be assigned to them by the Committee.

Dr. H. H. Mills suggested the issue of a circular to the medical profession calling attention to this matter. Many doctors had not come on the panel because they were afraid of having a number of people assigned to them. He understood that only about 1,000 to 1,100 members of the profession were likely to be available, and if any proportion of these could be persuaded to join the panel it would be advantageous.

Dr. Lauriston E. Shaw moved, and Dr. B. A. Richmond seconded, that a circular on these lines be issued, and this was agreed to.

At the same meeting Dr. H. H. Mills said that at the end of the year there were forty resignations, while forty-six practitioners came into the service. This was satisfactory because it conformed with the progress of the panels for the preceding months, and the Chairman, Mr. Dawes, said the doctors were still coming on.

